# The cognition of dynamic evaluation of operating room environmental quality among medical staff: a qualitative study

**DOI:** 10.3389/fpubh.2026.1950404

**Published:** 2026-10-15

**Authors:** Yanan Li, Tong Zhang, Dongmei Bian

**Affiliations:** Department of Surgery Operating Room, Xijing Hospital, Fourth Military Medical University, Xian, China

**Keywords:** environmental indicators, environmental monitoring, healthcare professionals' perception, operating room environment, qualitative research

## Abstract

**Objective:**

This qualitative study, guided by the Donabedian three-dimensional quality model, aimed to explore the real perceptions of multidisciplinary healthcare professionals regarding the dynamic evaluation of the operating room environment quality. The ultimate goal was to identify current management deficiencies and propose evidence-based strategies for optimization.

**Method:**

Using a descriptive qualitative design with a hybrid analytic orientation, purposive sampling recruited 7 operating-room nurses, 6 surgeons, 5 anesthesiologists, and 2 infection-control specialists from 4 tertiary Grade A hospitals. After ethics approval, formal semi-structured interviews were conducted in March 2026. Interview data were transcribed, manually coded, and analyzed in NVivo 15.0 using Colaizzi's method. The Donabedian model informed the interview guide and later organization of evaluation-related categories.

**Results:**

Three core themes were extracted: (1) the four-dimensional connotation of operating-room environmental quality, comprising physical, chemical, biological, and humanistic dimensions; (2) nine preliminary candidate indicators for dynamic evaluation, grouped across structure, process, and outcome domains; and (3) healthcare professionals' perceptions of potential safety and health implications for staff and patients. These findings highlight a gap between current static monitoring practices and the dynamic nature of intraoperative environmental risks.

**Conclusion:**

Participants' accounts suggest that a preliminary candidate framework for dynamic evaluation could incorporate structural conditions, process monitoring, and outcome-related feedback. Integrated management that considers both occupational health and patient safety represents a practice implication to be empirically evaluated. This study provides a qualitative foundation for subsequent Delphi consultation and psychometric or empirical validation of a dynamic OR environmental assessment framework.

## 1 Introduction

The operating room (OR) constitutes a critical setting for therapeutic interventions and emergency resuscitation, demanding rigorous standards to ensure the safe and efficient execution of surgical procedures (1, 2). Environmental quality within the OR is closely associated with both patient prognosis and the occupational safety of healthcare personnel (2, 3). Existing literature categorizes the OR environment into physical, chemical, and biological domains, encompassing key parameters such as architectural layout, environmental surfaces, temperature and relative humidity, air cleanliness, and ambient noise levels (4–6). Specific attention has been directed toward microbiological air control and the impact of acoustic environments on clinical outcomes, while broader human factors and environmental interactions continue to be refined through consensus guidance and systematic reviews (2, 4–7).

Presently, the monitoring of OR environmental quality predominantly relies on static hygienic indicators, including airborne bacterial colony counts and surface microbial contamination (2, 5, 8, 9). While these metrics offer insights into the OR's environmental status under static or at-rest conditions, they inherently fall short in representing the dynamic environmental shifts that occur during actual clinical activity. For instance, noise levels in the OR are not constant; they fluctuate significantly with surgical stages and personnel activities. Grant et al. (10) demonstrated that high noise levels are associated with increased communication failures, which may indirectly compromise patient safety. Furthermore, Boyle et al. (2) systematically reviewed environmental factors and found that noise negatively impacts operative team performance and is objectively associated with an increased risk of postoperative complications, including surgical site infections (SSIs). Similarly, waste anesthetic gases (WAGs) exhibit concentration variations throughout the surgical procedure, influenced by induction phases, equipment leaks, and ventilation efficiency. Keller et al. (11) noted that while real-time monitoring technologies exist, there is a lack of combined strategies that can simultaneously assess compliance with 8-h occupational exposure limits and identify short-term exposure peaks. Silva et al. (12) and Zhang et al. (13) further emphasized that without effective scavenging systems, WAG concentrations can exceed recommended thresholds during surgery, posing continuous occupational exposure risks. Additionally, indoor microbial loads are directly correlated with personnel movement and door opening frequencies, as simulated by Taaffe et al. (14), indicating that static sampling fails to capture these transient pollution events. Consequently, a sole reliance on static monitoring proves inadequate for comprehensively assessing intraoperative environmental risks. This deficiency may hinder the timely identification of hazardous conditions, thereby potentially contributing to elevated incidences of SSIs and increased occupational exposure incidents among healthcare personnel.

Despite the prevailing focus in research on the quantitative detection of general physicochemical indicators, a dedicated and comprehensive dynamic environmental quality assessment system for ORs remains absent. The “Three-Dimensional Quality Structure” model (Donabedian model) offers a robust theoretical framework for the comprehensive evaluation of medical service quality across three dimensions: structure, process, and outcome (15). An evaluation system constructed upon this model enables the systematic assessment of pertinent operational quality and efficiency (16–18). Dynamic environmental governance also depends on perioperative management arrangements. Staffing continuity, role allocation, workflow coordination, equipment readiness, and the organization of supporting services shape how consistently environmental protocols can be implemented and how quickly teams can respond to intraoperative variation (19). These managerial conditions fit naturally within the structural and process dimensions of the Donabedian model and provide a broader operational rationale for examining environmental quality from a multidisciplinary perspective. Therefore, guided by the principles of the Donabedian model, this study systematically delineated the multifaceted connotations of the OR environment and identified key elements for dynamic assessment. The delineation was approached from a multi-stakeholder perspective, involving OR nurses, surgeons, anesthesiologists, and infection-control specialists. Furthermore, this research critically analyzed perceived environment-related safety hazards, aiming to bridge the current research gap in intraoperative dynamic environmental assessment. By doing so, it seeks to provide a qualitative foundation for the prospective development and validation of a dynamic evaluation framework and refined, closed-loop management strategies for the OR environment.

## 2 Materials and methods

### 2.1 Design and participants

This study used a descriptive qualitative design with a hybrid analytic orientation. The Donabedian structure-process-outcome model was used deductively to inform the interview guide and provide a sensitizing framework for interpretation, whereas significant statements, meaning units, and initial codes were derived inductively from the interview transcripts using Colaizzi's seven-step method (20). The framework was therefore used to organize and interpret, rather than predetermine, the findings. Purposive sampling, guided by maximum variation, was used to recruit operating-room practitioners from four tertiary Grade A hospitals in different geographic regions of China: Affiliated Xijing Hospital of the Fourth Military Medical University, Affiliated Jinling Hospital of Nanjing Medical University, Affiliated Strait Hospital of Huaqiao University, and the 942nd Hospital of the Joint Logistics Support Force. Semi-structured interviews included OR nurses, surgeons, anesthesiologists, and infection-control specialists. The initial target sample size was 20 to 30 participants, and the final sample was determined by data saturation (21, 22). After ethics approval in March 2026, 22 eligible professionals were purposively approached through targeted invitations in the four hospitals. Eligible professionals were identified and contacted through the operating-room head nurses and department directors of each hospital. Two declined participation because their clinical workloads conflicted with the scheduled interview times, and 20 completed all study procedures. The final sample comprised 11 participants from Hospital A, 3 from Hospital B, 5 from Hospital C, and 1 from Hospital D.

Eligibility criteria were: (1) nurses had at least 5 years of operating-room work experience, were engaged in operating-room quality management, held a junior college degree or above, and held an intermediate professional title or above; (2) surgeons and anesthesiologists had at least 2 years of surgical practice experience, held a bachelor's degree or above, and held an intermediate professional title or above; (3) infection control specialists had at least 5 years of experience in infection control or a related field, held a bachelor's degree or above, and held an associate senior professional title or above; and (4) participants provided written informed consent and voluntarily agreed to complete an audio-recorded interview.

Exclusion criteria included: (1) Individuals who were absent from duty or unable to communicate one-on-one; (2) Those who declined full audio recording; (3) Participants who withdrew midway and were unable to complete the entire interview process.

To maximize the heterogeneity of sample information and broaden the scope of interview data, participants were stratified and selected across six dimensions: affiliated hospital, job position, length of service, educational background (Bachelor's / Master's / Doctoral degree), professional rank (Intermediate / Associate Senior / Senior), and gender.

### 2.2 Data collection

This qualitative study used semi-structured interviews. All formal interviews were conducted in March 2026 after ethics approval. Eight interviews were conducted face to face and 12 by telephone, according to participant preference and logistical feasibility. The same semi-structured guide, interviewer preparation, interview sequence, audio-recording procedures, and probing techniques were used across modes to support consistency.

#### 2.2.1 Development of the interview guide

Guided by the research question and the Donabedian structure-process-outcome model, the interview guide was drafted through literature review, research-group discussion, and expert consultation. Two pilot interviews were conducted after ethics approval. Because no changes to the interview guide were required, these interviews were retained in the final analysis. The interview questions were: (1) Regarding the operating room environment, what aspects do you believe constitute its overall composition? (2) From the perspectives of structure, process, and outcome, what dimensions should be considered when evaluating the dynamic environmental quality of the operating room? (3) What specific indicators do you consider relevant for evaluating the dynamic environmental quality of the operating room? (4) In your opinion, what adverse experiences does the current operating room environment impose on healthcare professionals or patients? Please provide some examples.

#### 2.2.2 Data collection procedures

Before participation, all individuals received information about the study objectives and procedures, provided written informed consent, and arranged an interview time. Interviews were conducted individually in a private, soundproofed room or by telephone, with no non-participants present in either mode. The full interview was audio-recorded, and field notes were made when necessary to record key information and non-verbal expressions in face-to-face interviews. Each interview lasted approximately 17 min on average (341 recorded minutes in total across the 20 interviews). Each participant was interviewed once; no repeat interviews were conducted (COREQ item 18). Interviewers were master's students specializing in operating-room research and held registered-nurse professional titles. Before each interview, participants were informed that the interviewer was a graduate student conducting the study as part of her research training. They had received coursework and training in qualitative interviewing and had no prior relationship with the participants. To promote reflexivity, the research team discussed their operating-room experience and assumptions about environmental quality before data collection, used the shared interview guide and neutral probes during interviews, and independently analyzed the data before resolving differences through discussion. Data collection stopped after interview 20 because no new codes or themes emerged after interview 18 and this finding was confirmed in two consecutive additional interviews.

### 2.3 Data analysis

All interview recordings were transcribed within 24 h to preserve data integrity and minimize recall bias. Each transcript was returned to the corresponding participant for review, and no substantive disagreements regarding wording or meaning were reported. Transcripts were imported into NVivo 15.0 for data management, manual coding, analysis, and categorization. Twenty interviews, totaling 341 min, generated 68,195 words of transcript and 452 initial codes. Data were analyzed using Colaizzi's seven-step method. In keeping with the hybrid design, researchers first identified significant statements and formulated meaning units inductively. For example, the statement “Personnel flow affects microbial concentration; personnel management must be strengthened” was interpreted as the meaning unit that personnel movement was perceived to influence environmental microbial risk; it was initially coded as “personnel flow management,” grouped under the category “personnel allocation and management,” and then organized within the structure-quality domain. The Donabedian model was used at the later stage to organize related categories within structure, process, and outcome, while preserving inductively generated codes and categories. The steps were: (1) reading all interview records; (2) identifying significant statements; (3) formulating meanings and coding recurrent viewpoints; (4) aggregating codes into categories; (5) producing a detailed description; (6) formulating themes; and (7) returning findings to participants for validation (23).

Coding followed a hybrid framework. A preliminary codebook was derived deductively from the Donabedian structure, process, and outcome domains and was extended with inductive codes generated from the transcripts. Two researchers independently coded each transcript in Nvivo 15.0 and compared their code sets after every four to five interviews. Disagreements were resolved by returning to the transcript and discussing the interpretation until consensus was reached; when consensus could not be reached, a third researcher acted as arbiter, and all coding decisions were recorded in a written audit trail. Codes were then grouped into categories by shared meaning, and categories were consolidated into candidate themes through repeated within-case and across-case comparison. The example above traces this chain from a participant statement to a meaning unit, a code, a category, and finally a structure-domain element, and further worked examples are held in the audit trail. Saturation was appraised separately for each professional group and for each Donabedian domain: no new codes emerged within any group after interview 18, and the two additional interviews confirmed that no new codes or themes appeared in any group. The audit trail records code definitions, category and theme decisions, and the rationale for merging or discarding candidate codes, and is available from the corresponding author on request.

### 2.4 Quality control

All research-team members completed training in qualitative research methods and interview communication skills and passed the relevant assessments. Interviews and transcription were conducted by designated personnel, and transcription was completed within 24 h after each interview. Two researchers independently analyzed the data and resolved differences through group discussion. Preliminary findings were returned to all 20 participants for member validation; each participant reviewed the themes, sub-themes, and supporting quotations. Where feedback identified ambiguous wording, the corresponding category or sub-theme description was clarified, and no participant proposed a change to the three themes or their structure. Interviews were conducted in Mandarin Chinese, and recordings were transcribed verbatim in simplified Chinese. Quotations presented in English were translated by the first author; translation accuracy and semantic equivalence were reviewed and confirmed through research-team discussion. By reporting the interview guide, the hybrid coding framework, the audit trail, and the analytic decisions, the study supports procedural replication, that is, comparable procedures that allow another team to repeat the approach rather than to obtain identical findings.

### 2.5 Ethical approval

Study preparation, including research design, literature review, and interview-guide development, occurred from November 2025 to February 2026. The study received ethics approval from the Ethics Committee of Xijing Hospital on 10 March 2026 (KY20262085-C-1) before recruitment and formal interviews began in March 2026.

## 3 Results

### 3.1 Characteristics of participants

Formal qualitative interviews were conducted in March 2026 with operating-room healthcare professionals and infection-control specialists from four tertiary Grade A hospitals. After interview 18, no new codes or themes emerged; this determination was confirmed in two consecutive additional interviews. Twenty participants were included: 7 operating-room nurses, 6 surgeons, 5 anesthesiologists, and 2 infection-control specialists. The mean age was 40.05 ± 6.65 years (SD), and the mean length of service was 16.30 ± 9.94 years (SD; range, 3–34 years). Eight participants held a bachelor's degree, 6 a master's degree, and 6 a doctoral degree. Fourteen participants held intermediate titles, 5 associate senior titles, and 1 a senior title. The sample included 11 men and 9 women. Detailed demographic information is shown in Table 1.

**Table 1:** General information of participants.

Personnel category	Code	Gender	Length of service (years)	Professional title	Age	Educational background
Surgeon	S1	Male	5	Intermediate	35	Doctoral degree
	S2	Male	5	Intermediate	30	Doctoral degree
	S3	Male	16	Associate Senior	43	Doctoral degree
	S4	Male	6	Intermediate	34	Master's degree
	S5	Male	12	Intermediate	35	Master's degree
	S6	Male	3	Intermediate	33	Doctoral degree
Anesthesiologist	A1	Male	15	Intermediate	39	Master's degree
	A2	Male	6	Associate Senior	36	Doctoral degree
	A3	Female	11	Intermediate	37	Doctoral degree
	A4	Male	16	Associate Senior	39	Master's degree
	A5	Male	3	Intermediate	30	Master's degree
Operating room NURSE	N1	Female	24	Intermediate	45	Bachelor's degree
	N2	Female	25	Intermediate	43	Bachelor's degree
	N3	Female	27	Intermediate	45	Bachelor's degree
	N5	Female	21	Intermediate	40	Bachelor's degree
	N6	Female	28	Intermediate	49	Bachelor's degree
	N7	Male	18	Intermediate	39	Bachelor's degree
Infection control specialist	I1	Female	33	Associate Senior	53	Master's degree
	I2	Female	34	Senior	52	Bachelor's degree

### 3.2 Theme extraction and analysis

Through coding in Nvivo 15.0 and analysis using Colaizzi's method, three major themes were extracted. Although the interview guide was informed by the Donabedian model, initial codes and categories were developed from participants' accounts; the model was subsequently used to organize the evaluation-related categories. A summary is provided in Table 2, followed by detailed thematic descriptions.

**Table 2:** Interview results.

Theme	Sub-themes	Category
The four-dimensional connotation of operating room environmental quality	The physical environment is the foundation of the operating room environment.	/
	The chemical environment is gaining increasing attention.	/
	The biological environment is the core of nosocomial infection and occupational protection.	/
	The humanistic environment addresses the dual needs of medical staff and patients.	/
Preliminary candidate indicators for dynamic evaluation of the operating room environment based on the “Three-Dimensional Quality Structure” model	Evaluation of structure quality	Structure and layout
		Policies and regulations
		Personnel allocation and management
	Evaluation of process quality	Personnel safety
		Personnel comfort
		Infection control
	Evaluation of outcome quality	Satisfaction evaluation
		Incidence of adverse events
		Qualification rate of objective environmental parameter indicators
Healthcare professionals' perceptions of potential safety and health implications	Perceived disruption of surgical work	/
	Perceived patient implications	/
	Perceived effects on healthcare professionals' physical and mental health	/

#### 3.2.1 Theme one: the four-dimensional connotation of operating room environmental quality

(1) The physical environment is the foundation of the operating room environment.

A1 stated: “The most intuitive aspect of the operating room environment is the physical environment, which includes not only temperature, ventilation, lighting, and noise but also the internal functional layout.” N7 further elaborated: “The physical environment of the operating room includes the layout of the operating room, temperature and humidity, lighting, as well as the types and quantities of instruments and equipment, etc.” I2 emphasized its structural necessity: “The operating room is a physical structure; it must have premises and a built environment, and provide a certain amount of space.”

(2) The chemical environment is gaining increasing attention.

I2 and N5 noted: “To ensure the cleanliness of the operating room, chemical disinfectants are frequently used to disinfect the air and environmental surfaces.” S1 added: “Pharmaceuticals and disinfectants—these chemical substances are also an aspect of the environment.”

(3) The biological environment is the core of nosocomial infection and occupational protection.

N2, I2, and S1 shared the view: “Electrosurgical instruments used during surgery generate smoke. Taking tumor surgery as an example, the smoke contains not only toxic gases produced by tissue incision but also aerosols formed by tumor tissues, which may cause harm to personnel in the room.”

(4) The humanistic environment addresses the dual needs of medical staff and patients.

N7 stated: “Preoperative reassurance and education, intraoperative warming, privacy protection, etc., represent humanistic care for patients and concern their surgical experience.” S1, S4, S5, and S6 collectively highlighted: “The atmosphere of doctor-nurse collaboration, teamwork, doctor-patient communication, and the management of work stress affect the mental and psychological state of medical staff.”

#### 3.2.2 Theme two: nine preliminary candidate indicators for dynamic evaluation of the operating-room environment

The nine preliminary candidate indicators are presented systematically in three Donabedian domains: structure quality, including (1) structure and layout, (2) policies and regulations, and (3) personnel allocation and management; process quality, including (4) personnel safety, (5) personnel comfort, and (6) infection control; and outcome quality, including (7) satisfaction evaluation, (8) adverse-event occurrence, and (9) the qualification rate of objective environmental parameters. The following excerpts are grouped under each candidate indicator.

(1) Evaluation of Structure Quality: This dimension focused on foundational elements.

① Structure and Layout: Most interviewees pointed out that the structure and layout of the OR must comply with regulations and facilitate efficient work. I1 noted: “The environmental layout of the operating room and the placement of items and equipment in the operating room need to be reasonably set up.” N7 added: “The placement position of instruments and equipment needs to be conducive to the efficient conduct of work.”

② Policies and Regulations: Strict protocols for cleaning, disinfection, occupational protection, environmental monitoring, and emergency plans were deemed essential. S6 stated: “The operating room should establish sound systems for nosocomial infection control and aseptic inspection, and clarify individual job responsibilities.” A5 mentioned: “Work systems should be displayed on the wall, including relatively fixed workflows and a stable structure of surgical personnel.” N2 and S4 affirmed: “The operating room environment must comply with national management standards as well as hospital and departmental regulations.”

③ Personnel Allocation and Management: Interviewees believed personnel quantity, activities, and standardized operations significantly impact OR environmental quality. N3 observed: “Personnel flow affects microbial concentration; personnel management must be strengthened.” S2, S4, S5, S6, A4, and A5 collectively suggested: “The operating room should have corresponding requirements for human resource allocation, manage personnel entry qualifications effectively, and control the number of personnel to reduce the impact of personnel activities or non-standard operations on the environment.”

(2) Evaluation of Process Quality: This dimension focused on operational aspects and safety.

① Personnel Safety: Most interviewees believed that safety hazards exist in the operating room environment, which is an issue that must be prioritized to improve environmental quality. I1 stated: “Many people do not wear laser safety glasses when using CO_2_ lasers, and our operating tables occasionally leak electricity; these are all safety hazards.” A1 noted: “The smoke produced by electrosurgical units irritates the respiratory tract of medical staff, and ordinary surgical masks cannot provide completely effective protection.”

② Personnel Comfort: Interviewees believed that the operating room environment can sometimes cause discomfort, affecting the progress of surgery to a certain extent. A2 commented: “The noise in the operating room, such as sounds from electrosurgical units, staff conversations, and instrument alarms, makes people feel uncomfortable.” N4 noted: “Without violating relevant regulations, the operating room environment could make some ‘warm' changes to alleviate the anxiety of patients and their families.” S1 emphasized: “For surgery to proceed smoothly and safely, participating surgical staff should maintain a good physical and psychological state, for example, through smooth intraoperative communication and a harmonious team atmosphere.”

③ Infection Control: Most interviewees believed that the quality of the operating room environment is closely related to surgical site infections. N4 asserted: “The surgical environment must comply with infection control standards.” A5 added: “The air quality in the operating room is very important for preventing surgical site infections in patients.”

(3) Evaluation of Outcome Quality: This dimension focused on results and feedback.

① Satisfaction Evaluation: Interviewees believed that the satisfaction of patients and medical staff plays an important role in the improvement and enhancement of environmental quality. S4, S5, and N7 agreed: “The satisfaction scores of medical staff and patients regarding the quality of the operating room environment are important evaluation indicators, reflecting the subjective experience of the environment.”

② Incidence of Adverse Events: Many participants emphasized the need to focus on preventing the occurrence of adverse events. S6 noted: “Outcome quality mainly includes the incidence rate of surgical site infections, the incidence rate of occupational exposure, and the incidence rate of intraoperative adverse events caused by environmental factors.” I1 remarked: “Currently, there are deficiencies in the self-protection of operating room staff; many people are unaware of the risk factors present in the environment. If they possessed knowledge in this area, many instances of occupational exposure could be reduced.”

③ Qualification Rate of Objective Environmental Parameter Indicators: infection control specialists proposed that whether environmental parameters meet the standards is the most direct and objective method for evaluating environmental quality. I1 suggested: “The laminar flow equipment and filters in the operating room need to be replaced regularly to ensure that relevant parameter indicators meet the standards before and after surgery, thereby ensuring personnel safety.” I2 added: “Purification system parameters should be treated as an outcome indicator; the air exchange rate and fresh air volume must meet requirements. Secondly, the dynamic qualification rate of intraoperative indicators is very important; key intraoperative time nodes can be selected for real-time monitoring.”

#### 3.2.3 Theme three: Healthcare professionals' perceptions of potential safety and health implications

This theme reflects healthcare professionals' perceptions and second-hand observations. The study did not interview patients or measure patient-level clinical outcomes; therefore, the following accounts should not be interpreted as evidence of causal effects on infection, recovery, prognosis, or other patient outcomes.

(1) Perceived Disruption of Surgical Work: Many surgeons described environmental conditions that they perceived could disrupt workflow or create safety concerns. S2 noted: “Various cables in the operating room are cluttered along activity paths. It is easy for personnel to trip or disconnect the cables when walking or changing positions during surgery, affecting the surgical process and safety.” S6 mentioned: “The noise from intraoperative instruments causes significant interference to doctors, easily causing distraction and delaying surgical progress.” S2 and S4 also highlighted: “The performance of the shadowless lamp in the room is unstable; sometimes it is too glaring, and sometimes the surgical area cannot be seen clearly.”

(2) Perceived Patient Implications: Participants perceived that unstable temperature and thermal discomfort might be relevant to patient comfort and postoperative recovery. These views reflect professional perceptions rather than direct patient reports or measured clinical outcomes. A1 stated: “The temperature in the operating room is sometimes unstable, which easily increases the risk of postoperative infection for patients.” A3, N1, N3, and N4 shared: “Due to nervousness or physical weakness, patients may feel that the operating room is very cold. Many patients also mentioned this issue during postoperative follow-ups, which prolongs the patient's recovery time.”

(3) Perceived Effects on Healthcare Professionals' Physical and Mental Health: Interviewees described potential implications of environmental quality for their physical and mental health. S1 and S6 observed: “Orthopedic surgeries are physically demanding. Sometimes when the operating room temperature is a bit high, I sweat profusely, which makes me feel a bit irritable.” N5 noted: “Scrub nurses stare at the surgical field for long periods, causing soreness and discomfort in their eyes.” N3 mentioned: “When some pediatric patients undergo inhalation anesthesia, the mask isn't sealed tightly, and we can smell the anesthetic gas while operating beside the patient.” N4 described: “The smell of smoke produced by electrosurgical units often fills the room. Sometimes, after the operating room is cleaned, a strong smell of disinfectant lingers.” N5 added: “When doctors fix specimens, formaldehyde leaks out, and the smell is very unpleasant.” S1 questioned: “Whether aerosols formed by tumor tissues might cause certain harm to operating room personnel.”

## 4 Discussion

### 4.1 Establishing a holistic and multidimensional paradigm for operating room environmental governance

Recent studies indicate a shift in healthcare professionals' perception of OR environmental quality, moving beyond conventional, unidimensional frameworks. The modern OR is now conceptualized as a four-dimensional, integrated perioperative ecosystem—encompassing physical, chemical, biological, and psychosocial determinants that interact synergistically to modulate overall environmental quality. While physical, chemical, and biological parameters remain the core targets of regulatory compliance, current protocols exhibit a disproportionate emphasis on patient-centric infection prevention and control (IPC), often overlooking occupational exposure risks to chemical and biological hazards faced by perioperative staff. Furthermore, environmental mitigation strategies predominantly rely on preoperative static sanitization and postoperative terminal disinfection, largely neglecting dynamic intraoperative environmental surveillance. The psychosocial environment, including ergonomic comfort and team dynamics, functions as a qualitative extension directly impacting patient experience and clinical staff performance, yet it remains consistently under-assessed in conventional environmental audits (24, 25).

The high-acuity clinical workload, interdisciplinary communication friction, and chronic high-stress clinical milieu frequently precipitate adverse psychological sequelae, such as irritability and anxiety, thereby exacerbating occupational burnout. Comprehensive psychosocial interventions can address this by ameliorating patient preoperative anxiety and creating psychological safety among staff (26). By optimizing interdisciplinary team synergies, regulating shift durations, and standardizing provider-patient communication pathways, these measures safeguard the physical and psychological well being of both patients and healthcare providers (27).

With the increasing prevalence of intelligent operating suites, the integration of advanced medical equipment and digital workflows has significantly heightened the complexity of intraoperative environments (28–30). Consequently, compartmentalized and static environmental management paradigms are no longer sufficient to meet contemporary occupational health and safety (OHS) standards or support the dynamic needs of next-generation surgical teams. Digital operating suites may help bridge static assessment and intraoperative feedback by integrating time-stamped data from environmental sensors, ventilation and purification systems, anesthesia workstations, surgical-smoke evacuation devices, and workflow records into a shared dashboard. Such a system could display selected parameter trends, identify deviations from locally defined thresholds, and prompt the responsible team to review conditions at key intraoperative time points (28). This study did not evaluate any specific technology; therefore, this pathway should be understood as a practical implementation hypothesis derived from the candidate framework, rather than as evidence of effectiveness.

Several dynamic monitoring approaches already exist in perioperative practice, including continuous particle and microbiological air sampling, real-time waste-anesthetic-gas sensing, noise dosimetry, and networked sensors that stream ventilation and purification parameters (11, 13, 28–30). Their limitations are nonetheless instructive. Most capture only a single parameter; air-sampling results are surrogate measures that do not map directly onto wound contamination or clinical outcomes; validated intraoperative thresholds remain scarce; and issues of calibration, cost, alarm burden, and data integration restrict routine use. Recognizing these approaches and their constraints clarifies that the candidate framework is intended to organize existing signals into a dynamic assessment structure rather than to introduce an entirely new monitoring technology.

For nurse managers and hospital administrators, institutionalizing dynamic indicators would require more than deploying sensors or dashboards. Practical challenges include selecting a feasible core set of indicators, defining ownership of data review and escalation, integrating records across devices and information systems, minimizing alarm burden and documentation workload, ensuring staff training and adherence, protecting data governance and privacy, and allocating resources for equipment maintenance and continuous quality review. A phased implementation beginning with a small, clinically meaningful indicator set and iterative feedback from multidisciplinary users may help establish local feasibility before wider adoption.

Therefore, it is imperative to establish a multidimensional and holistic environmental governance framework to fundamentally shift institutional paradigms regarding environmental stewardship. Furthermore, we recommend implementing mandatory perioperative environmental compliance training for all personnel entering the OR suite, reinforced with annual continuing education and competency-based assessments. By operationalizing theoretical frameworks and institutionalizing accountability, the overarching objective is to cultivate a culture where every healthcare worker serves as an active environmental steward.

### 4.2 Preliminary candidate framework for dynamic operating-room environmental evaluation

Participants' accounts suggest that current operating-room environmental evaluation is often perceived as focused on static or at-rest parameters and infection prevention. They also described intraoperative variation associated with personnel movement and equipment operation. These findings support the development and testing of a multi-level candidate framework for dynamic evaluation; they do not establish that static monitoring is ineffective or that a particular framework will mitigate dynamic risks.

The Donabedian model provided a useful organizing framework for the candidate structure-, process-, and outcome-related categories. Structural conditions may include layout, equipment and supplies, ventilation and purification systems, protective materials, entry and exit protocols, and environmental-hazard monitoring. Process-related candidates may include personnel-traffic management, surgical-smoke evacuation, waste-anesthetic-gas scavenging, noise control, personal protective equipment, and temperature and humidity management. Outcome-related candidates may include satisfaction, occupational exposure events, environmental-parameter compliance, and patient-related measures where valid data are available. These candidate elements require content validation, prioritization, feasibility assessment, reliability testing, threshold development, and empirical evaluation before they can be treated as an assessment system or used to infer intervention effects.

### 4.3 Practice implications: integrated attention to healthcare professionals and patients

Participants described environment-related concerns affecting both healthcare professionals and patients. These accounts support an integrated practice perspective that considers occupational health alongside patient safety. Because this study did not measure patient outcomes or intervention effects, this perspective should be regarded as a practice implication and hypothesis for future evaluation rather than as a demonstrated dual-protection effect.

Ventilation and purification systems, disinfection workflows, personnel-traffic management, noise reduction, temperature and humidity regulation, and humanistic care were identified by participants as areas for management attention. Future studies should test whether and under what conditions these strategies improve environmental parameters, occupational exposures, patient experience, or clinical outcomes. Integrating occupational-health considerations into operating-room environmental management may provide a useful direction for developing and evaluating future interventions.

## 5 Conclusion

Guided by the Donabedian model, this qualitative study elicited healthcare professionals‘ accounts of physical, chemical, biological, and humanistic dimensions of operating-room environmental quality. The structure-, process-, and outcome-related categories constitute preliminary candidate elements for future dynamic evaluation, rather than an established assessment system or a validated set of indicators. Participants' perspectives suggest practice implications for attention to equipment maintenance, risk prevention and control, and humanistic care; the effects of particular management strategies on surgical-site infection, occupational exposure, recovery, or other outcomes require empirical testing. Future work should use Delphi consultation, content-validity assessment, feasibility testing, reliability evaluation, threshold development, and empirical validation before any indicator system is implemented or used for performance assessment.

### 5.1 Limitations

All participants were recruited from tertiary Grade A hospitals, and the eligibility criteria favored experienced professionals with intermediate or senior titles. The sample may therefore underrepresent junior staff and perspectives from primary or lower-level hospitals, limiting transferability to other healthcare settings and systems. The Donabedian model informed the interview guide and later organization of evaluation-related categories; despite inductive coding and reflexive discussions, this framework and the researchers' operating-room backgrounds may have influenced theme generation and interpretation. Patients were not interviewed, and patient-level clinical outcomes were not measured. Patient-related findings therefore represent healthcare professionals' perceptions or second-hand observations. The use of face-to-face and telephone interviews may also have influenced the depth or nature of responses. Finally, this qualitative study generated candidate evaluation elements only; it did not assess content validity, weighting, feasibility, reliability, thresholds, discriminative ability, or other measurement properties. Future research should include broader settings and patient perspectives and use Delphi consultation and psychometric or empirical testing to develop and evaluate a feasible dynamic assessment framework.

## Data Availability

The raw data supporting the conclusions of this article will be made available by the authors, without undue reservation.

## References

[1] Humphreys H Bak A Ridgway E Wilson APR Vos MC Woodhead K . Rituals and behaviours in the operating theatre - joint guidelines of the healthcare infection society and the european society of clinical microbiology and infectious diseases. J Hosp Infect. (2023) 140:161–5. doi: 10.1016/j.jhin.2023.06.009 37454912

[2] Boyle CP Crichton J Sgrò A Michael SH Wigmore SJ Skipworth RJE . Impact of environmental factors on operative team performance: systematic review and guidance for optimising clinical practice. Surg Endosc. (2025) 39:7983–99. doi: 10.1007/s00464-025-12362-4 41219560 PMC12708707

[3] Nyberg A Jirwe M Fagerdahl A Otten V Haney M Olofsson B. Perioperative patient safety indicators-a delphi study. J Clin Nurs. (2025) 34:1351–63. doi: 10.1111/jocn.17212 38757741 PMC11933514

[4] Unyimeituk Erikanderson Gama MNB Carlskoog. Noise perception, sensitivity, and patient outcomes during cesarean delivery. Anesthesiol Res Pract. (2025) 2025:5707084. doi: 10.1155/anrp/5707084 40230812 PMC11996264

[5] Humphreys H Thomas AM Staniforth K Mackintosh CA Taylor GP Townsend R . Microbiological factors in the design, validation and verification of operating theatre ventilation: healthcare infection society consensus guidance for infection prevention and control professionals, and members of ventilation safety groups. J Hosp Infect. (2026) 175:333–56. doi: 10.1016/j.jhin.2026.06.021 42413827

[6] Gradisnik L Bunc G Ravnik J Velnar T. Enhancing surgical safety: microbiological air control in operating theatres at university medical centre maribor. Diagnostics. (2024) 14:1054. doi: 10.3390/diagnostics14101054 38786351 PMC11120459

[7] Lomax S Catchpole K Sutcliffe J. Human factors in anaesthetic practice. Part 2: clinical implications BJA Educ. (2024) 24:68–74. doi: 10.1016/j.bjae.2023.11.003 38304073 PMC10829084

[8] Xie A Sax H Daodu O Alam L Sultan M Rock C . Environmental cleaning and disinfection in the operating room: a systematic scoping review through a human factors and systems engineering lens. Infect Control Hosp Epidemiol. (2024) 45:10–1. doi: 10.1017/ice.2023.280 38477015 PMC11439593

[9] Tomazin R Matos T. Mycological methods for routine air sampling and interpretation of results in operating theaters. Diagnostics. (2024) 14:288. doi: 10.3390/diagnostics14030288 38337804 PMC10855394

[10] Grant L Nicholson P Davidson B Finch S Manias E. Communication failures and the influence of noise in the operating room: a prospective cohort study. J Clin Nurs. (2026) 35:3123–33. doi: 10.1111/jocn.70280 41810721

[11] Keller M Cattaneo A Spinazzè A Carrozzo L Campagnolo D Rovelli S . Occupational exposure to halogenated anaesthetic gases in hospitals: a systematic review of methods and techniques to assess air concentration levels. Int J Environ Res Public Health. (2023) 20:514. doi: 10.3390/ijerph20010514 36612837 PMC9819674

[12] Silva MAP Carvalho LIM Destro MV Braz LG Braz MG. From indoors to outdoors: impact of waste anesthetic gases on occupationally exposed professionals and related environmental hazards - a narrative review and update. Environ Toxicol Pharmacol. (2025) 113:104624. doi: 10.1016/j.etap.2024.104624 39732353

[13] Zhang X Yang Y Liu L Sun J Zeng Y Yang Z . Branch enhanced photoacoustic sensor for comprehensive sevoflurane monitoring. ACS Sens. (2025) 10:2598–608. doi: 10.1021/acssensors.4c03063 39927729

[14] Taaffe KM Allen RW Fredendall LD Shehan M Stachnik MG Smith T . Simulating the effects of operating room staff movement and door opening policies on microbial load. Infect Control Hosp Epidemiol. (2021) 42:1071–5. doi: 10.1017/ice.2020.1359 33342455

[15] Donabedian A. The quality of care: how can it be assessed? JAMA. (1988) 260:1743–8. doi: 10.1001/jama.1988.03410120089033 3045356

[16] Yang J Liu F Yang C Wei J Ma Y Xu L . Application of donabedian three-dimensional model in outpatient care quality: a scoping review. J Nurs Manag. (2025) 2025:6893336. doi: 10.1155/jonm/6893336 40313787 PMC12045680

[17] Tossaint-Schoenmakers R Versluis A Chavannes N Talboom-Kamp E Kasteleyn M. The challenge of integrating ehealth into health care: systematic literature review of the donabedian model of structure, process, and outcome. J Med Internet Res. (2021) 23:e27180. doi: 10.2196/27180 33970123 PMC8145079

[18] Menon NS Sankar JP Faizal S Sankaranarayanan S. The operating theatre as a catalyst for quality care. BMC Health Serv Res. (2025) 25:996. doi: 10.1186/s12913-025-13202-w 40731011 PMC12308943

[19] Cenacchi C Giusti M Peghetti A Quirini S Tinelli F Giorgi S . Optimization of nursing staff standards in the perioperative settings of the irccs university hospital of bologna. An improvement project Front Public Health. (2025) 13:1601290. doi: 10.3389/fpubh.2025.1601290 40642247 PMC12241087

[20] Colaizzi PF. Psychological research as the phenomenologist views it. In:Valle RS King M, editors. Existential-Phenomenological Alternatives for Psychology. New York, NY: Oxford University Press (1978) p. 48–71.

[21] Hennink M Kaiser BN. Sample sizes for saturation in qualitative research: a systematic review of empirical tests. Soc Sci Med. (2022) 292:114523. doi: 10.1016/j.socscimed.2021.114523 34785096

[22] Squire CM Giombi KC Rupert DJ Amoozegar J Williams P. Determining an appropriate sample size for qualitative interviews to achieve true and near code saturation: secondary analysis of data. J Med Internet Res. (2024) 26:e52998. doi: 10.2196/52998 38980711 PMC11267098

[23] Yavuz B Koç G Yildiz GN Yüce Başaran HD Koçyigit D çiftçi B. Prevention of ventilator-associated pneumonia: intensive care nurses' perspectives and solution-oriented recommendations-a qualitative study. BMC Nurs. (2026) 25. doi: 10.1186/s12912-026-04766-x 42186053 PMC13386849

[24] Hicks CW Rosen M Hobson DB Ko C Wick EC. Improving safety and quality of care with enhanced teamwork through operating room briefings. JAMA Surg. (2014) 149:863–8. doi: 10.1001/jamasurg.2014.172 25006700

[25] Mah A Kalocsai C Alam F Karim A Larouche J Hallbeck MS . Making the operating room (or) work: sociomaterial study of ergonomics in the or. Br J Surg. (2026) 113. doi: 10.1093/bjs/znag083 42405509

[26] Ferrada P Jones JS. Psychological safety as infrastructure: from the operating room to system-level leadership. Surgery. (2026) 195:110218. doi: 10.1016/j.surg.2026.110218 42127779

[27] Ferreira RA Pereira SCDA Ribeiro OMPL Bueno AAB Pereira FCDA Henrique DM . Implementation strategies by leaders and health professionals to improve the safety climate in the operating room: a scoping review protocol. BMJ Open. (2025) 15:e94904. doi: 10.1136/bmjopen-2024-094904 40295131 PMC12039010

[28] Bellini V Russo M Domenichetti T Panizzi M Allai S Bignami EG. Artificial intelligence in operating room management. J Med Syst. (2024) 48:19. doi: 10.1007/s10916-024-02038-2 38353755 PMC10867065

[29] Azad TD Jopling JK Najjar P. Ambient artificial intelligence in the operating room: from passive recording to predictive coordination. Ann Surg. (2026). doi: 10.1097/SLA.0000000000007073 [Epub ahead of print]. 42026714

[30] Amato CL Yang C Badihi N Bernhard L Ratib O Seeliger B . Central command suite: futureproofing next-generation surgical environments to embrace the digital operating room. Int J Comput Assist Radiol Surg. (2025) 20:507–12. doi: 10.1007/s11548-024-03278-w 39708232 PMC11929672

